# Mobile phone survey estimates of perinatal mortality in Malawi: A comparison of data from truncated and full pregnancy histories

**DOI:** 10.1111/tmi.14109

**Published:** 2025-04-20

**Authors:** Georges Reniers, Julio Romero‐Prieto, Michael Chasukwa, Funny Muthema, Sarah Walters, Bruno Masquelier, Jethro Banda, Emmanuel Souza, Boniface Dulani

**Affiliations:** ^1^ Department of Population Health London School of Hygiene and Tropical Medicine London UK; ^2^ Institute of Public Opinion and Research Zomba Malawi; ^3^ Department of Politics and Government University of Malawi Zomba Malawi; ^4^ North‐West University, Afrocentric Governance of Public Affairs, Faculty of Humanities Potchefstroom South Africa; ^5^ Center for Demographic Research, UCLouvain Louvain‐la‐Neuve Belgium; ^6^ Malawi Epidemiology and Intervention Research Unit Lilongwe Malawi; ^7^ Department of Population Studies University of Malawi Zomba Malawi

**Keywords:** Malawi, Mobile phone surveys, perinatal mortality rate, stillbirth rate

## Abstract

**Objectives:**

In many low‐ and middle‐income countries, perinatal mortality estimates are derived retrospectively from periodically conducted household surveys. Mobile phone surveys offer advantages in terms of cost and ease of implementation. However, their suitability for monitoring perinatal mortality has not been established.

**Methods:**

We use data from the Malawi Rapid Mortality Mobile Phone Survey (RaMMPS) to estimate perinatal mortality rates from two versions of the survey instrument: a full pregnancy history and a shorter truncated pregnancy history. Female respondents of reproductive age were randomly allocated to either of these instruments. The sample was generated through random digit dialling with active strata monitoring. Post‐stratification weighting was used to correct for sample selection bias, and estimates are reported with bootstrap confidence intervals. We estimated the stillbirth rate as the synthetic cohort probability of a foetal death with 28+ weeks of gestation over all pregnancies reaching the same gestational age. The perinatal and extended perinatal mortality rates were defined as the probabilities of dying between 28 weeks and 7 or 28 days of life, respectively. RaMMPS estimates are compared to the 2015–2016 Malawi Demographic and Health Survey and estimates published by the United Nations Inter‐agency Group for Child Mortality Estimation.

**Results:**

Truncated and full pregnancy histories were administered for 2093 and 2067 women, respectively. Weighted point estimates of the stillbirth (19.81 deaths per 1000 pregnancies, 95%‐confidence interval (CI): 14.11–25.62), perinatal (42.41, 95%‐CI: 33.91–50.92), and extended perinatal mortality rates (50.11, 95%‐CI: 41.56–58.84) from the full pregnancy history instrument are in line with Demographic and Health Survey and United Nations Inter‐agency Group for Child Mortality Estimation estimates. In comparison, the mortality estimates from the truncated pregnancy history instrument are higher, but this difference only approaches statistical significance in the case of the stillbirth rate. Post‐stratification weighting produces a small upwards adjustment in the estimates.

**Conclusion:**

Mobile phone surveys are a promising method for collecting perinatal mortality data. The full pregnancy history instrument produces more plausible results than the shorter truncated pregnancy history questionnaire where the window of retrospection is restricted.

## INTRODUCTION AND BACKGROUND

The UN estimated that 1.9 million babies were stillborn in 2021, and 2.3 million children died in the first month of life [1]. Regional disparities in the burden of perinatal mortality are large, and recent progress in the reduction of stillbirths and neonatal mortality has been modest in comparison to maternal and post‐neonatal mortality. UNICEF labelled this *a neglected tragedy* [2]. It is also *an invisible tragedy* because most perinatal deaths, including stillbirths, occur in countries where routine administrative data (e.g., Civil Registration and Vital Statistics or Health Management Information Systems) are insufficiently performant to produce estimates that are useful for monitoring progress towards global targets [3, 4]. In these settings, perinatal mortality estimates are derived from periodically conducted household surveys, including the Demographic and Health Surveys.

Owing to the rapid expansion in mobile phone ownership, mobile phone surveys have become an appealing alternative to traditional household surveys. They can be deployed rapidly and without the need for in‐person contact—a key feature that makes them more suitable in the context of epidemic outbreaks or other humanitarian crisis situations [5]. Whereas mobile phone surveys are increasingly common [6, 7], they have not yet been used for measuring stillbirth and neonatal mortality. This study thus aims to ascertain whether plausible perinatal mortality estimates can be generated from a mobile phone survey, and complements another manuscript wherein we used mobile phone survey data to estimate infant and under‐five mortality [8].

There are several possible methodological pitfalls associated with mortality estimation from mobile phone surveys, including acceptability, sample selectivity, and data quality concerns [9, 10, 11, 12, 13]. One element that might affect acceptability and data quality is the duration of the interview in the sense that longer interviews are more susceptible to interruptions and the respondent's loss of concentration. The empirical evidence for the latter is not very strong [14], but as long as data quality can be upheld, short duration interviews are desirable for the mere reason that they reduce the burden on the respondent and fieldwork operational costs.

In this contribution, we use data from the Malawi *Rapid Mortality Mobile Phone Survey* (RaMMPS) where women of reproductive age were randomly administered one of two versions of a questionnaire module designed to measure stillbirth and neonatal mortality. One of the criteria on which they differed was the length of the module. The *full pregnancy history* questionnaire was adapted from the model Demographic and Health Surveys questionnaire that was introduced in 2020 (Demographic and Health Surveys round VIII), after it was established that it was better suited to identify stillbirths than full birth histories with a reproductive calendar (Demographic and Health Surveys round IV) and supplementary questions for identifying non‐live births (Demographic and Health Surveys round VII) [15, 16]. The full pregnancy history instrument elicits information about all pregnancies in chronological order, starting with the first pregnancy. We compare the full pregnancy history estimates of perinatal mortality with those from a *truncated pregnancy history* instrument, whereby the data is collected in reverse chronological order until an a priori defined date is reached. This questionnaire was modelled after truncated birth histories collected in Malaria Indicator Surveys. Owing to the truncation, the truncated pregnancy history instrument is shorter, making it potentially better suited for a mobile phone survey. Early methodological work evaluating instruments and the order in which birth or pregnancy histories are to be collected was not always conclusive, but a recent comparison of truncated versus full birth histories suggests that the former produces estimates of child mortality that are biased downwards [17, 18, 19, 20].

In the following sections, we describe the data and estimation procedures, and compare the Malawi RaMMPS estimates of the stillbirth and perinatal mortality rates from both questionnaires with estimates from the 2015–2016 Malawi Demographic and Health Survey and the UN Inter‐agency Group for Child Mortality Estimation [21, 22].

## DATA AND METHODS

### Data and survey instruments

We use data from the national Malawi RaMMPS conducted between 24 January 2022 and 28 July 2023. The fieldwork for this study was coordinated by the Institute of Public Opinion and Research (https://www.ipormw.org/) in Zomba, Malawi. The sample for the Malawi RaMMPS was generated via (screened) random digit dialling without replacement. Using the mobile phone numbering structure in Malawi, a set of random numbers was generated by Sample Solutions (https://sample.solutions/), and verified against the Home Location Register, which is a database of registered (including pre‐paid) numbers on the GSM network. The screening using the Home Location Register identifies the bulk of numbers that are not in use. Thereafter, trained enumerators conducted Computer Assisted Telephone Interviews with active strata monitoring [23]. Strata were a priori defined in terms of broad age groups (18–49 and 50–64), sex, region (North, Central, and South), and residential setting (urban/rural). Quotas for each combination of these attributes were derived from the 2018 census [24]. Once a quota was reached, respondents with these attributes were no longer eligible to participate in the study. Fieldwork was divided into four blocks of 4–5 months each, and quotas were re‐set at the beginning of each fieldwork block. Minors below the age of 18 were not interviewed to ensure that all respondents could consent to the interviews themselves. Respondents received 1200 Malawian Kwacha (~1.5 USD in 2021) in airtime as compensation for completing the interview. Enumerators worked from their homes, and a random sample of interviews was recorded (with consent) for quality control purposes. A fieldwork supervisor also conducted follow‐up calls with respondents who completed the interview.

In comparison to other sub‐Saharan African countries, mobile phone ownership in Malawi is relatively low. In 2021, the number of SIM cards per 100 individuals in Malawi was estimated at 60% and falls short of the sub‐Saharan African average, where mobile phone penetration was 93% [25]. Mobile phone ownership is particularly low in rural areas, where the gender gap in ownership is also more pronounced. According to the 2015–2016 Malawi Demographic and Health Surveys, 73.8% and 63.9% of men and women in urban areas owned a mobile phone. In rural areas, 47.1% and 25.9% of men and women owned a phone, respectively [26]. In addition, a large majority of the Malawian population lives in rural areas (84% according to the 2018 census [24]). Given these imbalances in population distribution and mobile phone ownership, enumerators had difficulty filling the quotas for rural respondents (women in particular), and this reduced the yield of completed Computer Assisted Telephone Interviews towards the end of each fieldwork block when the quotas for the easiest to reach respondents had been filled. To alleviate this, we fielded an interactive voice response (IVR) survey to identify rural respondents, and the details are described elsewhere [27].

Starting in the second fieldwork block (26 May 2022), consenting female respondents aged 18–49 were randomly allocated to the truncated pregnancy history or full pregnancy history set of questions (Supporting Information, SI‐1). The full pregnancy history questions were modelled on the instrument that was used in round VIII of Demographic and Health Surveys and solicited information on pregnancy dates, pregnancy outcomes, time of gestation, and the survival status of children [16]. As in the Demographic and Health Surveys, this detailed reproductive history was preceded by a summary pregnancy history to determine the total number of pregnancies for each woman.

Truncated pregnancy histories left censor pregnancies with an end date more than 7 years before the interview. Unlike the full pregnancy histories, truncated pregnancy histories were recorded in reverse chronological order, an approach that was adapted from the *Malaria Indicator Surveys*. To keep the interview as short as possible, the truncated pregnancy history instrument was not preceded by a set of summary pregnancy history questions. The truncated pregnancy history and full pregnancy history questionnaire modules used the same set of questions to collect information on the day of birth or pregnancy termination, as well as the gestational age.

For the purposes of the analyses presented here, the pregnancy history data were administratively (left) censored on 1 January 2014. Results with a left censoring date of 1 January 2016 are included as Supporting Information SI‐3.

### Post‐stratification weighting

Mortality estimates from MPS may be affected by selection bias because mobile phone ownership—and possibly also respondent consent—is correlated with respondent characteristics that have a bearing on mortality. The imposition of quotas for the a priori defined strata only partially alleviates this problem because these are limited to key demographic (age group and sex) and geographic (region and urban/rural place of residence) attributes. It is more challenging to impose quotas on educational background or wealth in a sample constituted via random digit dialling because their inclusion would considerably slow down fieldwork in the sense that it becomes much harder for enumerators to identify eligible respondents with the desired combination of attributes. We therefore resorted to post‐stratification to ensure that the RaMMPS sample is representative of the entire population in terms of a broader number of attributes, including education, household size, and household wealth.

Weights were estimated by Iterative Proportional Fitting—also known as raking; a method that is regularly used in mobile phone surveys [28]. For each fieldwork block, univariate distributions of the RaMMPS data were matched to the female population aged 18–49 in the household roster of the 2015–2016 Malawi Demographic and Health Survey, which was the most recent nationally representative survey available at the time of writing. Weights were computed and applied for the following attributes: (i) age group (18–29, 30–39, or 40–49); (ii) urban versus rural place of residence; (iii) region (northern, central, or southern); (iv) educational attainment (incomplete primary or less, completed primary and incomplete secondary, completed secondary or higher); (*v*) household size (1–4, 5–8, or 9+); and (vi) an indicator variable for household‐level access to a source of electricity. Our weighting procedure comes with two caveats: (i) the Demographic and Health Survey distribution of the weighting variables are themselves characterised by stochasticity (albeit small, see Table 1); and (ii) raking only reproduces the marginal distributions for each of the weighting variables in the reference dataset. Normalised weights ranged from 0.02 to 12.45 with a mean value of 1.0. Untrimmed weights are used in the manuscript, as this is sometimes recommended for small samples [29, 30]. Estimates with trimmed weights are included as Supplementary Material SI‐3b.

**TABLE 1:** Background characteristics of female respondents aged 18–49 in RaMMPS (weighted and unweighted) and the 2015–2016 Malawi Demographic and Health Survey (all women and the subset of mobile phone owners).

	TPH		FPH		DHS VII, women 18–49	
Attributes	Weighted	Unweighted	Weighted	Unweighted	All	m. owner
Place of residence: urban	18.82	37.17	17.83	37.78	18.41	35.41
	[*17.22*, *20.50*]	[*35.09*, *39.32*]	[*16.13*, *19.45*]	[*35.61*, *39.96*]	[*18.16*, *18.64*]	[*34.36*, *36.35*]
Region: North	12.42	17.06	10.89	16.50	11.68	15.04
	[*10.99*, *13.76*]	[*15.48*, *18.71*]	[*9.63*, *12.24*]	[*14.90*, *18.14*]	[*11.54*, *11.83*]	[*14.44*, *15.61*]
Central	40.99	36.50	44.36	37.45	42.85	40.71
	[*38.84*, *43.07*]	[*34.50*, *38.46*]	[*42.24*, *46.44*]	[*35.22*, *39.38*]	[*42.57*, *43.15*]	[*39.56*, *41.75*]
South	46.63	46.44	44.85	46.11	45.47	44.29
	[*44.53*, *48.71*]	[*44.34*, *48.64*]	[*42.60*, *46.90*]	[*44.07*, *48.33*]	[*45.17*, *45.75*]	[*43.23*, *45.27*]
Age: 18–29	50.22	56.90	54.14	58.10	52.41	50.07
	[*48.06*, *52.32*]	[*54.85*, *59.08*]	[*51.74*, *56.22*]	[*56.02*, *60.04*]	[*51.61*, *53.22*]	[*48.65*, *51.38*]
30–39	33.13	30.53	28.83	28.74	30.80	34.11
	[*31.06*, *35.28*]	[*28.71*, *32.35*]	[*26.95*, *31.01*]	[*26.92*, *30.82*]	[*30.08*, *31.58*]	[*32.71*, *35.53*]
40–49	16.72	12.52	17.03	13.16	16.79	15.83
	[*15.15*, *18.35*]	[*11.16*, *14.05*]	[*15.34*, *18.72*]	[*11.80*, *14.51*]	[*16.18*, *17.43*]	[*14.82*, *16.87*]
Education: less than complete primary	64.60	14.72	65.36	14.27	64.65	42.04
	[*62.42*, *66.60*]	[*13.33*, *16.41*]	[*63.28*, *67.27*]	[*12.77*, *15.87*]	[*63.98*, *65.34*]	[*40.76*, *43.46*]
Incomplete secondary	24.61	29.91	24.58	30.67	24.75	33.35
	[*22.77*, *26.56*]	[*27.95*, *31.89*]	[*22.81*, *26.54*]	[*28.81*, *32.80*]	[*24.04*, *25.42*]	[*31.96*, *34.58*]
Complete secondary or more	10.80	55.33	10.01	55.06	10.61	24.63
	[*9.56*, *12.04*]	[*53.30*, *57.60*]	[*8.81*, *11.37*]	[*52.78*, *57.14*]	[*10.06*, *11.09*]	[*23.35*, *25.86*]
Household size: 1–4	37.74	35.26	42.62	38.80	40.46	41.28
	[*35.69*, *39.94*]	[*33.06*, *37.36*]	[*40.47*, *44.61*]	[*36.77*, *40.88*]	[*39.63*, *41.28*]	[*39.87*, *42.63*]
5–8	55.14	54.71	50.90	52.10	52.74	52.06
	[*52.75*, *57.21*]	[*52.60*, *56.78*]	[*48.72*, *53.12*]	[*49.98*, *54.28*]	[*51.94*, *53.62*]	[*50.75*, *53.49*]
9+	7.12	10.03	6.48	9.05	6.80	6.67
	[*6.02*, *8.27*]	[*8.74*, *11.42*]	[*5.52*, *7.62*]	[*7.84*, *10.38*]	[*6.40*, *7.20*]	[*6.00*, *7.43*]
Electricity access	14.05	52.27	13.06	53.60	13.74	30.26
	[*12.61*, *15.65*]	[*50.07*, *54.35*]	[*11.76*, *14.59*]	[*51.38*, *55.85*]	[*13.29*, *14.19*]	[*29.16*, *31.40*]
Observations	2093	2093	2067	2067	21,392	8151

*Note*: Both RaMMPS and Demographic and Health Survey (DHS) estimates are reported with bootstrapped 95%‐percentile CIs and report the median value of the bootstrap distribution as the point estimate. For the DHS, women were resampled within the same cluster. The distribution of background characteristics for the DHS is given for all women aged 18–49 (the reference for computing the post‐stratification weights) and the subset of mobile phone owners.

Abbreviations: FPH, full pregnancy histories; TPH, truncated pregnancy histories.

### Stillbirth and perinatal mortality rate estimation

We calculated the‐late‐stillbirth rate from the RaMMPS data as the synthetic cohort probability of a pregnancy loss with at least 28 weeks of gestation, $q\left(28w,\text{Birth}\right)$, and is reported as the number of stillbirths per 1000 live and stillbirths combined. The perinatal mortality rate is defined analogously but expands the exposure time to the first week of life: $q\left(28\;\text{weeks},7\;\text{days}\right)$. For analytical purposes, we also present the extended perinatal mortality rate, covering the first 28 days of life: $q\left(28\;\text{weeks},28\;\text{days}\right)$. The latter circumvents the problem of heaping at 7 days and allows us to compare RaMMPS with UN Inter‐agency Group for Child Mortality Estimation estimates. UN Inter‐agency Group for Child Mortality Estimation estimates are available for the stillbirth rate and the neonatal mortality rate, but no estimates are published for the probability of dying during the first week of life (i.e., the early neonatal mortality rate). UN Inter‐agency Group for Child Mortality Estimation estimates for the perinatal mortality rate are computed by the authors, assuming a log‐normal distribution of these indicators.

To facilitate direct comparisons with the RaMMPS data, the Demographic and Health Survey sample was restricted to women aged 18–49. Furthermore, we extracted the date of pregnancy termination (month and year; the day was imputed) from the Demographic and Health Survey reproductive calendar as this information is not included in the full birth history instrument itself. UN Inter‐agency Group for Child Mortality Estimation estimates are reported for all women of reproductive age, including adolescents aged 15–17, not included in the RaMMPS data.

Confidence intervals (CIs) for RaMMPS estimates were computed via nonparametric bootstrapping, resampling the total number of interviews 1,000 times with replacement. For each bootstrap sample, probabilities of selection were proportional to the post‐stratification weights and inversely proportional to the probability of inclusion in the interviewed set of respondents. The 50th percentile is reported as the central tendency of these distributions; and the 2.5th and 97.5th percentiles were used to report 95% CIs and to test for statistical significance. CIs for the Demographic and Health Survey data are produced using a similar procedure. UN Inter‐agency Group for Child Mortality Estimation estimates are reported with 90% confidence bounds.

## RESULTS

Out of the 56,072 mobile phone numbers that were tried, a RaMMPS Computer Assisted Telephone interview was completed with 13,800 respondents (men and women aged 18–64). Response and refusal rates were 26.55% and 10.42%, respectively. These are defined as the number of completed interviews or refusals over the number of respondents who either met the inclusion criteria, or whose eligibility for inclusion in the study could not be established. As described elsewhere, this response rate is a lower bound estimate because cases with unknown eligibility are included in the denominator [31]. The analyses in the remainder of this manuscript are restricted to 4160 complete interviews with women aged 18–49 (after listwise deletion of 52 cases with missing information on background variables used for weighting). About half of these women were administered a truncated pregnancy history and about half received the full pregnancy history instrument (SI‐2). The median duration to administer the full pregnancy history instrument (including summary pregnancy history questions) was 3.20 min (Q1–Q3: 0.80–5.18). The median duration to administer the truncated pregnancy history instrument (not including summary pregnancy history questions) was 2.07 min (Q1–Q3: 0.38–3.10).

Table 1 provides the individual and household attributes of the RaMMPS respondents in the two pregnancy history modules and the Demographic and Health Survey reference dataset. For the RaMMPS data, we present estimates before and after post‐stratification weighting. Demographic and Health Survey estimates are given for all female respondents aged 18–49 and for the subgroup of women who own a mobile phone. The Demographic and Health Survey data confirm that mobile phone owners are more frequently urban, better educated, and more often have access to electricity. This is also reflected in the distribution of these attributes in the unweighted RaMMPS samples (columns 2 and 4). Malawi RaMMPS respondents also appear to come from slightly larger households, and that may be due to the fact that larger households have a greater likelihood of being sampled via random digit dialling, or that estimated household sizes are biased upwards in RaMMPS. After weighting (columns 1 and 3), the imbalance in the RaMMPS data is largely rectified, and the marginal distribution of these background characteristics matches that in the Demographic and Health Survey sample for all women (column 6).

**FIGURE 1:**
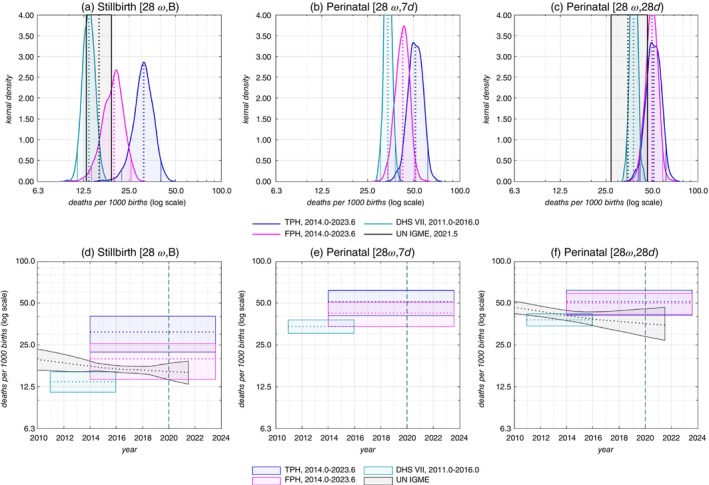
Stillbirth and perinatal mortality estimates in the Malawi RaMMPS (by survey instrument) compared with UN Inter‐agency Group for Child Mortality Estimation (UN‐IGME) and Demographic and Health Survey (DHS) estimates. Panels (a)–(c) contain the bootstrap distributions of the RaMMPS and DHS perinatal mortality estimates along with estimates from UN‐IGME. In panels (d)–(f), the same estimates are plotted on a time scale and are restricted to the 95%‐bootstrap CIs. RaMMPS and DHS estimates pertain to all women aged 18–49. The UN‐IGME estimates in panels (c) and (f) have been computed by the authors using estimates of stillbirth ratios and the neonatal mortality rate, and assuming a log‐normal distribution of these parameters. UN‐IGME does not publish separate mortality estimates for the first 7 days of life, and it is not possible to compute the UN‐IGME equivalent for the indicator in panels (b) and (e). UN‐IGME estimates are reported with 90% uncertainty bounds. TPH, truncated pregnancy histories; FPH, full pregnancy histories.

Figure 1 contains two representations of the stillbirth and perinatal mortality indicators. The top row (panels (a)–(c)) compares the bootstrapping distribution of the estimates from the two RaMMPS pregnancy history modules along with the UN IGME estimates and the 2011–2016 Demographic and Health Surveys estimates. The bottom row of Figure 1 (panels (d)–(f)) shows the same estimates on a time scale. Figure 1 contains estimates after post‐stratification. Both weighted and unweighted estimates are reported in Table 2. As Supplementary Information SI‐4, we also provide perinatal mortality estimates by the background characteristics that are used for post‐stratification weighting. Unless stated differently, we refer to weighted RaMMPS estimates in the text.

**TABLE 2:** Malawi stillbirth and perinatal mortality rates by source, before and after post‐stratification weighting.

	Stillbirth [28 weeks, B)		Perinatal [28 weeks, 7 days)		Perinatal [28 weeks, 28 days)	
Source	Weighted	Unweighted	Weighted	Unweighted	Weighted	Unweighted
TPH, 2014.0–2023.6	31.02	24.89	51.32	44.41	51.42	44.99
	[*22.23*, *40.23*]	[*17.21*, *33.30*]	[*40.70*, *61.81*]	[*33.70*, *55.86*]	[*40.87*, *61.97*]	[*34.29*, *56.30*]
FPH, 2014.0–2023.6	19.81	16.52	42.41	40.66	50.11	45.03
	[*14.11*, *25.62*]	[*10.59*, *23.18*]	[*33.91*, *50.92*]	[*31.51*, *51.68*]	[*41.56*, *58.84*]	[*35.45*, *56.01*]
DHS VII, 2011.0–2016.0	13.56		33.87		37.89	
Women 18–49	[*11.41*, *16.00*]		[*30.38*, *37.85*]		[*34.27*, *41.97*]	
DHS VII, 2011.0–2016.0	15.66	17.10	35.89	38.77	38.58	42.03
Women 18–49, mobile owners	[*10.72*, *21.37*]	[*11.55*, *22.64*]	[*28.21*, *43.86*]	[*31.06*, *46.50*]	[*30.86*, *47.24*]	[*33.81*, *50.42*]
UN‐IGME, 2019.5	16.15				35.97	
	[*14.44*, *18.10*]				[*29.44*, *45.25*]	
UN‐IGME, 2020.5	16.00				35.29	
	[*13.66*, *18.68*]				[*28.14*, *45.84*]	
UN‐IGME, 2021.5	15.80				34.81	
	[*13.04*, *19.06*]				[*27.01*, *46.83*]	

*Note*: DHS estimates were computed for all women aged 18–49, and for the subset of mobile phone owners before and after post‐stratification weighting. Both RaMMPS and DHS estimates are reported with bootstrapped 95%‐percentile CIs and report the median value of the bootstrap distribution as the point estimate. UN Inter‐agency Group for Child Mortality Estimation (UN IGME) estimates of perinatal mortality are computed by the authors from the stillbirth mortality rate and the neonatal mortality rate.

Abbreviations: TPH, truncated pregnancy histories; FPH, full pregnancy histories.

**FIGURE 2:**
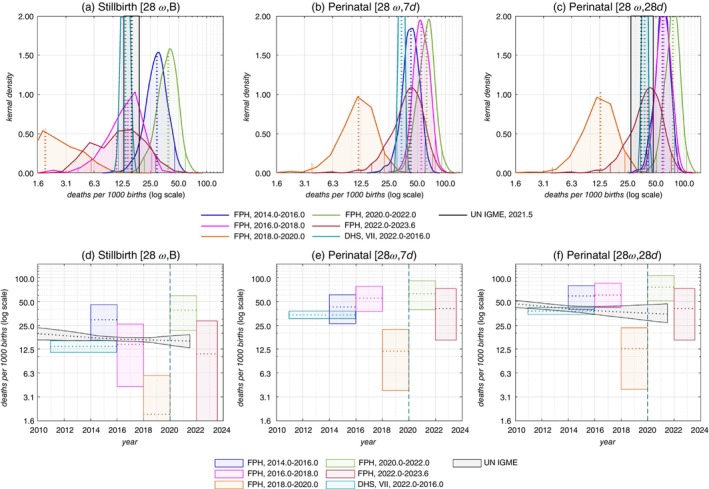
Stillbirth and perinatal mortality rates in the Malawi RaMMPS by period (full pregnancy history (FPH) instrument only) compared with UN IGME and DHS estimates. See Figure 1.

The RaMMPS full pregnancy history estimate for the stillbirth rate (19.81 per 1000, 95%‐CI: 14.11–25.62) is comparable to that of the UN Inter‐agency Group for Child Mortality Estimation estimate for 2019 (16.15, 90%‐CI: 14.44–18.10). The Demographic and Health Survey estimate is lower, but the difference is not statistically significant. It is also worth noting that the 2015–2016 Malawi Demographic and Health Survey used the full birth history instrument along with a reproductive calendar, and this is now considered to be inferior to a full pregnancy history questionnaire for capturing stillbirths [15]. The RaMMPS truncated pregnancy history estimate (31.02, 95%‐CI: 22.23–40.23) is considerably higher.

The RaMMPS full pregnancy history estimates of perinatal mortality (42.41 per 1000, 95%‐CI: 33.91–50.92) and extended perinatal mortality rate (50.11, 95%‐CI: 41.56–58.84) exceed estimates from other published sources, but the differences are again not statistically significant. The 2019 UN Inter‐agency Group for Child Mortality Estimation estimate for the extended perinatal mortality rate, for example, is 35.97 (90%‐CI: 29.44–45.25).

Post‐stratification weighting produces a modest upwards adjustment in the RaMMPS perinatal mortality estimates, but they are never significantly different from the unweighted estimates. Application of the post‐stratification weighting procedure produces a small downwards correction to the perinatal mortality estimates from the subsample of mobile phone owners from the Demographic and Health Surveys. Again, the CIs of the weighted, unweighted, and full samples overlap.

Figure 2 (SI‐5) contains stillbirth and perinatal mortality estimates from the RaMMPS full pregnancy history instrument disaggregated by 2‐year intervals. Point estimates are indicative of a mortality decline between 2014 and 2020 for each of the indicators that are considered, but they are also suggestive of a temporary mortality reversal in the calendar years corresponding with the COVID‐19 outbreak (i.e., 2020–2022). The uncertainty around the period‐specific full pregnancy history estimates is, however, large. Furthermore, the mortality estimates for the period just before and after 2020–2022 are very low. While this may result from the stochastic variation in perinatal deaths, it is also possible that there is some displacement of events that artificially inflates the mortality estimates for 2020–2022.

One of the complications with evaluating perinatal mortality data and estimates is the absence of a gold standard measurement for most high mortality settings. As done elsewhere, we therefore revert to an evaluation of the age patterns of mortality to ascertain whether stillbirth and early childhood mortality estimates are plausible. In Table 3, this is done in terms of two ratios: (i) the stillbirth to neonatal mortality rate ratio; and (ii) the early neonatal to neonatal mortality rate ratio.

**TABLE 3:** Data quality checks: Perinatal mortality rate ratios.

	Stillbirth [28 weeks, B)		ENMR q (7 days)		NMR q (28 days)		Stillbirth/NMR		ENMR/NMR	
Instrument/method	Weighted	Unweighted	Weighted	Unweighted	Weighted	Unweighted	Weighted	Unweighted	Weighted	Unweighted
TPH, 2014.0–2023.6	31.02	24.89	20.94	19.98	21.03	20.62	1.47	1.20	1.00	0.97
	[*22.23*, *40.23*]	[*17.21*, *33.30*]	[*14.67*, *28.22*]	[*13.01*, *27.37*]	[*14.67*, *28.22*]	[*13.58*, *28.12*]	[*0.96*, *2.30*]	[*0.78*, *2.01*]	[*0.97*, *1.00*]	[*0.89*, *1.00*]
FPH, 2014.0–2023.6	19.81	16.52	22.98	24.76	31.01	29.14	0.63	0.57	0.74	0.86
	[*14.11*, *25.62*]	[*10.59*, *23.18*]	[*16.39*, *29.65*]	[*17.37*, *32.69*]	[*23.46*, *38.45*]	[*21.32*, *37.74*]	[*0.42*, *0.95*]	[*0.34*, *0.91*]	[*0.63*, *0.85*]	[*0.74*, *0.95*]
DHS VII, 2011.0–2016.0	13.56		20.59		24.74		0.55		0.83	
Women 18–49	[*11.41*, *16.00*]		[*18.15*, *23.49*]		[*21.92*, *27.99*]		[*0.44*, *0.67*]		[*0.79*, *0.88*]	
DHS VII, 2011.0–2016.0	15.66	17.10	20.22	22.07	23.12	25.35	0.68	0.67	0.88	0.87
Women 18–49, mobile owners	[*10.72*, *21.37*]	[*11.55*, *22.64*]	[*14.59*, *26.73*]	[*16.48*, *28.34*]	[*17.44*, *29.65*]	[*19.37*, *31.87*]	[*0.43*, *1.03*]	[*0.43*, *1.00*]	[*0.80*, *0.94*]	[*0.80*, *0.93*]

*Note*: 95% Bootstrap confidence intervals.

Abbreviations: ENMR, early neonatal mortality rate; FPH, full pregnancy histories; NMR, neonatal mortality rate; TPH, truncated pregnancy histories.

The stillbirth to neonatal mortality rate ratio from the full pregnancy history instrument in the Malawi RaMMPS (0.63, 95%‐CI: 0.42–0.95) is on par with the 2015–2016 Malawi Demographic and Health Surveys. Median values for the stillbirth to neonatal mortality rate across Demographic and Health Survey rounds (all countries) with birth/pregnancy histories range from 0.43 to 0.67 [16]. Estimates of the ratio from population‐based prospectively collected data in low‐ and middle‐income countries are somewhat higher: 0.83 (95%‐CI: 0.78–0.89) [32]. The truncated pregnancy history instrument estimate of the stillbirth to neonatal mortality rate ratio is considerably higher than those that are reported elsewhere (1.47, 95%‐CI: 0.96–2.30).

The early neonatal mortality rate to neonatal mortality rate ratio from the full pregnancy history instrument in the Malawi RaMMPS (0.74, 95%‐CI: 0.63–0.85) also compares well to the Malawi Demographic and Health Survey estimates included in Table 3. Median estimates of this ratio across all Demographic and Health Survey rounds range from 0.69 to 0.81 [16]. Again, the truncated pregnancy history estimate is higher than estimates from other sources.

## DISCUSSION

We have used—for the first time—mobile phone survey data for estimating perinatal mortality via the truncated and full pregnancy history instruments. These questionnaires were adapted from those used in face‐to‐face surveys, and female respondents (aged 18–49) were randomly allocated to either of these two instruments.

The full pregnancy history instrument produces point estimates of the stillbirth (19.81, 95%‐CI; 14.11–25.62) and (extended) perinatal mortality rates (50.11, 95%‐CI: 41.56–58.84) that are comparable to those published by the UN Inter‐agency Group for Child Mortality Estimation and the 2015–2016 Malawi Demographic and Health Survey. The truncated pregnancy history estimate for the stillbirth rate is considerably higher and possibly less plausible. This is corroborated by the data quality checks in terms of the stillbirth to neonatal mortality rate ratio, which is uncharacteristically high for the truncated pregnancy history instrument. The same holds for the fraction of neonatal deaths that occur in the first week (i.e., the early neonatal mortality rate to neonatal mortality rate ratio). In contrast, the extended perinatal mortality rate estimates for both survey instruments are statistically equivalent and comparable to the Demographic and Health Survey and UN Inter‐agency Group for Child Mortality Estimation.

The time gained from administering the shorter truncated pregnancy history instrument—amounting to a difference in the median duration of just over 1 min—hardly justifies the use of the truncated survey instrument, so our results support the use of full pregnancy histories for measuring perinatal mortality in a mobile phone survey. Furthermore, it is worth noting that the time needed to collect pregnancy histories over the phone (typically less than 5 min) was considerably shorter than data collection in a face‐to‐face survey, for which a mean duration of around 10 min has been reported [15]. Whether this has repercussions for data quality could not be established, but it is certainly an element that requires further consideration. Factors that might contribute to shorter interview durations in a mobile phone survey are the lower fertility rates among mobile phone owners and differences in the conversational style in a telephone versus an in‐person interview.

Selection bias is a systemic problem in mobile phone surveys, and particularly so in circumstances where mobile phone ownership is not universal and possibly correlated with the outcomes of interest. To minimise or circumvent this problem, we have (i) used a quota sample with active strata monitoring and (ii) used post‐stratification weighting to ensure that our sample represents the population of interest on a number of socio‐demographic background characteristics. Because we imposed sampling quotas for urban and rural respondents, the application of post‐stratification weights produced a relatively small upwards adjustment in the perinatal mortality estimates only. As argued elsewhere, this approach seems suitable for correcting mobile phone‐based mortality estimates, but may be insufficient to recover population estimates for indicators (e.g., contraceptive use or fertility) that are an expression of preferences in addition to one's socio‐demographic attributes [11, 12].

This study was limited by its relatively small sample size. First, this was driven by the difficulty to identify and reach rural women; a problem that may be less pronounced in populations where mobile phone ownership is higher—and the gender divide in ownership is smaller—than in Malawi. Second, we introduced full pregnancy histories and the randomised comparison of both survey instruments only during the fourth month of fieldwork, following the publication of another study suggesting that shortened (a.k.a. *truncated*) instruments tend to produce biased mortality estimates of under‐five mortality [17]. In that study, the bias in under‐five mortality estimates from truncated ‘birth’ histories was typically downward, whereas the truncated pregnancy history instrument used in this study resulted in relatively high stillbirth estimates. Owing to the relatively small sample sizes in this study, it is not possible to draw firm conclusions about mortality differentials over time, or, mortality differentials by the respondent's background characteristics.

## CONCLUSION

Mobile phone surveys are a promising tool for collecting perinatal mortality data where birth and death registration is incomplete, and whenever an alternative to an in‐person survey is needed. In comparison to the truncated pregnancy history instrument, full pregnancy histories yield estimates of perinatal mortality levels and age patterns that align more closely with other sources. Given that the additional time needed to collect full pregnancy history is marginal, we are thus inclined to advocate their use in future mobile phone surveys. Adjudication between the two instruments for estimating perinatal mortality should, however, rest on confirmatory studies in larger samples.

## FUNDING INFORMATION

This study was made possible with financial support from the *Bill and Melinda Gates Foundation* (INV‐023211). The funder had no role in study design, data collection, data analysis, data interpretation, or writing of the report.

## CONSENT TO PARTICIPATE

All participants provided oral consent for the survey, including consent for storing anonymized data in a public repository and consent to audio record the interview.

## Supporting information


Data S1–S5

## Data Availability

The 2022–2023 Malawi RaMMPS data are publicly available from the DataFirst portal (https://doi.org/10.25828/M86Z-NF08). Code to reproduce the results is available from the GitHub repository maintained by JRP (https://github.com/Romero-Prieto/RaMMPS_Malawi).
